# A genetic variant near adaptor-related protein complex 2 alpha 2 subunit gene is associated with coronary artery disease in a Chinese population

**DOI:** 10.1186/s12872-018-0905-2

**Published:** 2018-08-07

**Authors:** Sibo Wang, Zhihui Ma, Yongjun Zhang, Yankui Ding, Zhong Chen, Liansheng Wang

**Affiliations:** 1 Department of Cardiology, the First Affiliated Hospital of Nanjing Medical University, Nanjing, 210029 China; 2 Department of Cardiology, Affiliated Sixth People’s Hospital East of Shanghai University of Medicine and Health Sciences, No. 222 Huanhu Xisan Road, Shanghai, 201306 China; 3 Shanghai Jiao Tong University Affiliated Sixth People’s Hospital, No. 222 Huanhu Xisan Road, Shanghai, 201306 China

**Keywords:** SNP, single nucleotide polymorphism, Association, rs7396366, rs2526378, CAD, coronary artery disease, Susceptibility

## Abstract

**Background:**

Adaptor-related protein complex 2 alpha 2 subunit (AP2A2) gene encodes a protein-a subunit of the AP-2 adaptor protein complex. Evidence has revealed that benzodiazepine receptor-associated protein 1 (BZRAP1) is abundant in the hippocampus with potential effects on brain diseases. Recently, an epidemiological study reported that two variants (rs7396366 and rs2526378) closest to the AP2A2 and BZRAP1 genes are associated with higher plasma lipids and Alzheimer’s disease. Whether the two single nucleotide polymorphisms (SNPs) are actually relevant to coronary artery disease (CAD) and CAD severity remains elusive. Our aim was to assess whether these two SNPs are relevant to CAD and its severity in a Chinese population.

**Methods:**

Three hundred and thirty-five patients with documented CAD (282 stable CAD, 28 non-ST-segment elevation myocardial infarction, 25 ST-segment elevation myocardial infarction), and 372 non-CAD controls were included in the study. The participants were divided into two groups according to coronary angiography results. CAD patients were further demarcated into subgroups with one-, two-, or three-vessel stenosis. Genotypes at rs7396366 and rs2526378 were examined using polymerase chain reaction-ligase detection reaction. The association between these two SNPs with CAD and its severity were analyzed.

**Results:**

The frequency of the rs7396366 TT genotype was significantly higher in CAD patients than in controls (13.7% vs. 7.8%, 95% CI: 1.15–3.07, *P* = 0.014). Subjects with a variant genotype T allele had an increased risk of CAD compared with G allele carriers (additive model: 95% CI: 1.21–3.35, *P* = 0.008). After adjustment for traditional cardiovascular risk factors, analysis of the dominant models involving rs7396366 also showed that T allele carriers had a significantly higher risk for CAD than G allele carriers had (dominant model: OR 1.48, 95% CI: 1.03–2.14, *P* = 0.035). Age, sex, type 2 diabetes mellitus, fasting plasma glucose, and the TT genotype in rs7396366 were significantly associated with three-vessel lesions. Despite these significant outcomes of rs7396366, information on rs2526378 showed no significant difference between CAD patients and non-CAD controls.

**Conclusion:**

Our results show that the T allele and TT genotype in rs7396366, closest to the AP2A2 gene, are linked to an increased risk of CAD and its severity in a Chinese population.

**Electronic supplementary material:**

The online version of this article (10.1186/s12872-018-0905-2) contains supplementary material, which is available to authorized users.

## Background

Cardiovascular disease (CVD) is a major cause of death worldwide and is primarily responsible for the loss of health throughout the world [1, 2]. Coronary artery disease (CAD), the most common CVD, remains the major cause of morbidity and mortality in the largest developing country, China [3, 4]. CAD is a complicated, multifactorial disease that involves both genetic and environmental factors [5]. In the past decade, genome-wide association studies (GWAS) have provided abundant evidence to help determine the quantity of susceptibility gene loci associated with CAD and its severity [6–10].

Adaptor-related protein complex 2 (AP2) is a protein complex that is involved in the formation of clathrin-coated pits and functions as an adaptor by linking lipid and protein membrane components with the clathrin lattice [11]. It has four non-identical polypeptide chains [12]. The AP2 subunit α2- (AP2A2) has been identified as playing a pivotal role in regulating clathrin-mediated endocytosis [13]. More recently, AP2 has evolved as a key regulatory node to coordinate clathrin-coated pits formation and ensure high spatial and temporal regulation of clathrin-mediated endocytosis [14].

Benzodiazepine receptor-associated protein 1 (BZRAP1), also known as PRAX-1 and rab-interacting molecule-binding protein 1 (RIM-BP1), is an 1857-amino acid protein encoded by the BZRAP1 gene. Previous studies have shown that BZRAP1 is prevalent in the mesolimbic system, especially abundant in the CA1 subfield of the hippocampus [15]. Scholars have observed the upregulation of BZRAP1 after chronic antidepressant treatment [16]. In addition, BZRAP1 mutations are demonstrated to be associated with autism-spectrum disorder and schizophrenia [17, 18].

Recently, an epidemiological study reported that two genetic variants of single nucleotide polymorphisms (SNPs), rs7396366, whose closest gene is AP2A2, and rs2526378, whose closest gene is BZRAP1, are associated with higher plasma lipids and Alzheimer’s disease [19].

Because both Alzheimer’s and CAD are metabolic-related and age-related diseases, we analyzed the potential relationship of rs7396366 and rs2526378 to CAD. However, as far as we know, no data to date have demonstrated the in-depth links between them. To advance our knowledge of the functions of AP2A2 and BZRAP1 in CAD and to seek new methods predicting CAD earlier, we studied the association between the two SNPs and the risk of CAD and its severity based on a Chinese population.

## Methods

### Participant samples

The study consisted of 707 independent individuals divided into two cohorts: 335 with CAD and 372 non-CAD controls. All of the participants were consecutively recruited from the Department of Cardiology, Shanghai Jiao Tong University Affiliated Sixth People’s Hospital East. Each study participant underwent cardiac catheterization for clinical assessment of CAD. CAD was defined as at least one main coronary artery with luminal narrowing > 50% (main coronary artery, left anterior descending branch, left circumflex branch, right coronary artery, and their large side branches). According to their clinical manifestations and auxiliary examinations, for example, electrocardiogram and serum myocardial injury markers during hospitalization, we divided CAD cases into stable CAD (SCAD), non-ST-segment elevation myocardial infarction (NSTEMI), and ST-segment elevation myocardial infarction (STEMI) subgroups. In addition, to reflect the severity of their coronary artery lesions, CAD samples were devided into one-, two-, and three-vessel lesion subgroups. Controls were defined as having no main coronary artery with luminal narrowing > 10%. The following patients were excluded from the study: those with congenital heart disease, renal failure, malignant tumors, and age ≤ 18 years. Flow chart see in Additional file 1.

### Ethics, consent and permissions

Approval of the Ethics Committee of Shanghai Jiao Tong University Affiliated Sixth People’s Hospital East was obtained before the start of the work, and each participant provided written informed consent.

### Clinical variables collection

Clinical variables in the study included age, sex, body mass index (BMI), smoking, hypertension, diabetes, total cholesterol and plasma glucose. Information on these variables was obtained from the participants and the hospital information system. Subjects were considered smokers if they were smoking at the time of sample collection and had a smoking history of at least half a year. Hypertension was defined as a systolic blood pressure ≥ 140 mmHg and/or a diastolic blood pressure ≥ 90 mmHg or taking any antihypertensive medications. Type 2 diabetes mellitus (T2DM) was identified based on blood sugar levels with fasting levels higher than 7.0 mmol/L and/or a random plasma glucose > 11.1 mmol/L or taking an antidiabetes medication.

### Genotype determination

After overnight fasting, together with blood collection for clinical variables, blood samples for genotype determination was also obtained. Serum and white blood cells was separated by centrifugation and stored at − 80° until further analysis. We identified the two SNPs’ (rs7396366 and rs2526378) genotypes by using the polymerase chain reaction-ligase detection reaction strategy described previously [20–22].

### Statistical analysis

SPSS software version 19.0 for Windows (SPSS Inc., Chicago Illinois, USA) was used for the statistical analysis. Continuous variables, such as age, BMI, total cholesterol level, and fasting plasma glucose (FPG), are expressed as the mean ± standard deviation (SD). The Student *t* test was used to examine the differences between two groups. For the analysis of Hardy-Weinberg equilibrium, the chi-square (χ^2^) test was used. Categorical variables including genetic frequencies, allelic frequencies, and the difference in genotypic and allelic frequencies among different groups and subgroups were also examined by χ^2^ analysis. We also used odds ratio (OR) and 95% confidence interval (CI) to find the relevance between the two genetic polymorphisms (G/T& C/T) and the risk of CAD and its severity. To determine whether AP2A2 or BZRAP1 genetic variants were independent of other clinical risk factors in predicting the severity of CAD, a logistic regression was used, adjusted by age, sex, smoking, hypertension, T2DM, total cholesterol level, and fasting glucose. More importantly, we utilized additive and dominant models to analyze the differences in the G/T or C/T polymorphism between CAD patients and controls. All data were considered statistically significant with a *P* value < 0.05 (two-tailed).

## Results

### Baseline characteristics

Tables 1 and 2 present the baseline characteristics of the study samples and the comparison between CAD, as well as its clinical subgroups, and controls for different risk factors. Compared with controls, patients in the CAD group had a significantly higher proportion of male gender, hypertension, T2DM and higher average values of age and FPG levels (all *P* < 0.01). However, data showed no significant difference between the two groups with regards to BMI, smoking, and total cholesterol level (*P* = 0.648, 0.221, 0.196, respectively). Furthermore, both SCAD and NSTEMI subgroups showed significant differences in age, sex, T2DM, and FPG levels compared with controls. However, a significant difference was only seen in age when STEMI subjects were compared with controls (*P* = 0.001). Based on the results of coronary angiography, one-, two-, three-vessel disease subgroups included 173 (51.6%), 85 (25.4%), and 77 (23.0%) patients, respectively.

**Table 1:** Baseline characteristics of CAD cases and non-CAD controls

Variables	CAD cases (*n* = 335)	Controls (*n* = 372)	*P* values
Age (years)	66.66 ± 11.00	58.11 ± 11.53	<.001
Sex (male), n (%)	196 (58.5)	175 (47.0)	.002
BMI (kg/m^2^)	25.24 ± 3.40	25.09 ± 4.39	.648
Smoking, n (%)	105(31.3)	101 (27.2)	.221
Hypertension, n (%)	245 (73.1)	217 (58.3)	<.001
Total cholesterol (mmol/L)	4.49 ± 1.24	4.64 ± 1.55	.196
T2DM, n (%)	92 (27.5)	58 (15.6)	<.001
Fasting plasma glucose (mmol/L)	6.06 ± 1.76	5.70 ± 1.62	.005
Number of vessels involved, n (%)			
one	173 (51.6)		
two	85 (25.4)		
three	77 (23.0)		

Abbreviations: *CAD* coronary artery disease, *BMI* body mass inde, *T2DM* type 2 diabetes mellitus

Age, BMI, total cholesterol and fasting plasma glucose are expressed as the mean ± SD and were compared using the Student *t* test. Other data are expressed as frequencies and percentages and were compared using the χ2-test

**Table 2:** Clinical characteristics in controls and CAD subgroups

Variables	CAD (*n* = 335) subgroups						Controls (*n* = 372)
	SCAD(*n* = 282)	*P*	NSTEMI(n = 28)	*P*	STEMI(*n* = 25)	*P*	
	*n* (%)		*n* (%)		*n* (%)		*n* (%)
Age (years)	66.44 ± 10.98	< 0.001	69.46 ± 11.21	< 0.001	66.04 ± 11.20	0.001	58.11 ± 11.53
Sex (male)	159 (56.4)	0.018	22 (78.6)	0.001	15 (60.0)	0.222	175 (47.0)
BMI (kg/m^2^)	25.53 ± 3.70	0.227	25.34 ± 4.37	0.826	23.07 ± 2.78	0.054	25.09 ± 4.39
Smoking, n (%)	80 (28.4)	0.730	15 (53.6)	0.005	10 (40.0)	0.172	101 (27.2)
Hypertension, n (%)	215 (76.2)	< 0.001	17 (60.7)	0.845	13 (46.4)	0.538	217 (58.3)
Total cholesterol(mmol/L)	4.49 ± 1.26	0.239	4.41 ± 1.14	0.464	4.51 ± 1.11	0.689	4.64 ± 1.55
T2DM, n (%)	71 (25.2)	0.002	11 (39.3)	0.003	10 (40.0)	0.004	58 (15.6)
Fasting plasma glucose (mmol/L)	5.97 (1.66)	0.038	6.40 ± 1.65	0.027	6.75 ± 2.65	0.061	5.70 ± 1.62
Number of vessels involved, n (%)							
one	163 (57.8)		6 (21.4)		4 (16.0)		
two	67 (27.8)		10 (35.7)		8 (32.0)		
three	52 (18.4)		12 (42.9)		13 (52.0)		

Abbreviations: *SCAD* stable coronary artery disease, *STEMI* ST-segment elevation myocardial infarction, *NSTEMI* non-ST-elevation myocardial infarction. Other abbreviations list in Table 1

Age, BMI, total cholesterol and fasting plasma glucose are expressed as the mean ± SD and were compared using the Student *t* test. Other data are expressed as frequencies and percentages and were compared using the χ2-test

### Analysis of genotype frequencies

Table 3 shows the genotype distribution of rs7396366 and rs2526378 in CAD patients and controls and the associated analysis. Deviation from the Hardy-Weinberg Equilibrium of the genotype distribution in the study was excluded (*P* > 0.05). A significantly higher proportion of rs7396366 TT genotype was present in CAD patients in comparison with that in controls (13.7% vs.7.8%, *P* = 0.014). The T genotype allele carriers of rs7396366 had a significantly higher risk of CAD than those with the G allele analyzed using the additive model (OR 2.02, 95% CI 1.21–3.35, *P* = 0.008). After controlling for the effects of traditional cardiovascular risk factors, analysis of the dominant model concerning rs7396366 also showed that the T allele compared with the G allele was associated with a significantly higher risk of CAD (dominant model: OR 1.48, 95% CI 1.03–2.14, *P* = 0.035). However, data for rs2526378 showed no significant difference following the same statistical methods except that the proportion of TT genotype was significantly higher in controls than in CAD patients (OR 1.43.95% CI 1.01–2.03, *P* = 0.044).

**Table 3:** Association analysis of rs7396366 and rs2526378 genotypes between CAD cases and controls

Genotypes	Control (*n* = 372)	CAD cases (*n* = 335)	OR (95% CI)	*P*	Multiple adjusted OR ^a^ (95% CI)	*P*
	*n* (%)	*n* (%)				
Rs7396366						
GG	206 (55.4)	162 (48.4)	0.76 (0.56–1.02)	.062	0.67 (0.47–0.97)	.035
GT	137 (36.8)	127 (37.9)	1.05 (0.77–1.42)	.766	1.27 (0.87–1.86)	.210
TT	29 (7.8)	46 (13.7)	1.88 (1.15–3.07)	.014	1.58 (0.86–2.89)	.142
Additive model			2.02 (1.21–3.35)	.008	1.82 (0.97–3.42)	.063
Dominant model			1.33 (0.99–1.78)	.062	1.48 (1.03–2.14)	.035
Additive model: TT vs. GG. Dominant model: (TT + GT) vs. GG						
Rs2526378						
CC	98 (26.3)	91 (27.2)	0.96 (0.69–1.34)	.806	1.02 (0.80–1.50)	.912
CT	157 (42.2)	155 (46.3)	0.85 (0.63–1.14)	.277	1.20 (0.85–1.69)	.295
TT	102 (27.4)	70 (20.9)	1.43 (1.01–2.03)	.044	0.74 (0.50–1.10)	.139
Additive model			1.35 (0.89–2.05)	.155	0.75 (0.44–1.27)	.282
Dominant model			1.07 (0.76–1.50)	.698	1.06 (0.70–1.60)	.797
Additive model: TT vs. CC. Dominant model: (TT + CT) vs. CC						

Abbreviations: *OR* odds ratio, *CI* confidence interval. Other abbreviations see in Table 1

^a^Logistic regression model, adjusted by age, sex, hypertension, diabetes, smoking, BMI, and fasting plasma glucose

### Gene-sex, gene-hypertension, and gene-smoking interactions

Further analysis to seek the effects of rs7396366 gene-sex, gene-hypertension, and gene-smoking interactions were conducted and the results were shown in Table 4. The data strongly suggest that male subjects with GT + TT genotypes had an increased risk of CAD (OR 1.88, 95% CI 1.32–2.67, *P* < 0.001). Subjects with GT + TT genotype and hypertension had a 3.01-fold higher risk of CAD (95% CI 2.12–4.28, *P* < 0.001). Additionally, subjects with GT + TT genotype and diabetes had a higher risk of CAD (OR 2.55, 95% CI 1.54–4.21, *P* < 0.001) than controls. After adjustment for other cardiovascular risk factors, the results remained unchanged. However, no evidence was found to identify higher risk of CAD in female subjects with GT + TT genotype, subjects with GT + TT genotype and diabetes, and subjects with GT + TT genotype and hypertension after adjustment (all *P* > 0.05).

**Table 4:** Gene-sex, gene-hypertension, and gene-diabetes interactions of rs7396366 in patients with CAD and controls

Variables		Controls	CAD cases	OR (95% CI)	*P*	Adjusted OR^a^ (95% CI)	*P*
		*n* (%)	*n* (%)				
Rs7396366	Sex						
GG	Female	100 (26.9)	65 (19.4)	0.66 (0.46–0.93)	.019	0.58 (0.37–0.91)	.018
GG	Male	106 (28.5)	97 (29.0)	1.02 (0.74–1.42)	.892	1.02 (0.67–1.54)	.929
GT + TT	Female	98 (26.3)	74 (22.1)	0.79 (0.56–1.12)	.188	0.75 (0.48–1.16)	.198
GT + TT	Male	68 (18.3)	99 (29.6)	1.88 (1.32–2.67)	<.001	2.29 (1.44–3.59)	<.001
Rs7396366	Hypertension						
GG	No	93 (25.0)	43 (12.8)	0.44 (0.30–0.66)	<.001	0.52 (0.32–0.84)	.007
GG	Yes	113 (30.4)	119 (35.5)	1.26 (0.92–1.73)	.146	0.97 (0.66–1.43)	.876
GT + TT	No	104 (28.0)	47 (14.0)	0.42 (0.29–0.62)	<.001	1.13 (0.68–1.89)	.641
GT + TT	Yes	62 (16.7)	126 (37.6)	3.01 (2.12–4.28)	<.001	1.50 (1.02–2.21)	.038
Rs7396366	T2DM						
GG	No	173 (46.5)	122 (36.4)	0.66 (0.49–0.89)	.007	0.68 (0.46–0.99)	.042
GG	Yes	33 (8.9)	40 (11.9)	1.39 (0.86–2.27)	.216	0.91 (0.50–1.64)	.748
GT + TT	No	141 (37.9)	121 (36.1)	0.93 (0.68–1.26)	.624	1.13 (0.77–1.65)	.545
GT + TT	Yes	25 (6.7)	52 (15.5)	2.55 (1.54–4.21)	<.001	2.33 (1.24–4.38)	.009

Abbreviations: *OR* odds ratio, *CI*, confidence interval

^a^Logistic regression model, adjusted by age, sex, hypertension, diabetes, smoking, BMI, and fasting plasma glucose

### The association between clinical risk factors and the severity of CAD

Table 5 presents the baseline clinical characteristics and rs7396366 genotype frequency among controls and the one-, two-, and three-vessel disease subgroup subjects. Age, sex, hypertension, total cholesterol level, T2DM, and fasting plasma glucose level were all statistically significantly different among the four groups (all *P* < 0.05), whereas the other variables showed no significant association (all *P* > 0.05). More importantly, the rs7396366 TT genotype also was significantly different among the four groups according to the χ^2^ test.

**Table 5:** Clinical characteristics in controls and CAD cases with different number of vessel lesions

Variables	Number of vessels involved				*P*
	0 (*n* = 372)	1 (*n* = 173)	2 (*n* = 85)	3 (*n* = 77)	
Age (years)	58.11 ± 11.53	65.67 ± 10.81	66.65 ± 11.29	68.90 ± 10.95	<.001
Sex (male)	175 (47.0)	88 (50.9)	58 (68.2)	53 (68.8)	<.001
BMI (kg/m^2^)	25.09 ± 4.39	25.25 ± 3.57	25.64 ± 3.93	24.84 ± 3.87	.356
Smoking, n (%)	101 (27.2)	47 (27.2)	29 (34.1)	29 (37.7)	.190
Hypertension, n (%)	217 (58.3)	125 (72.3)	66 (77.6)	54 (79.1)	<.001
Total cholesterol(mmol/L)	4.64 ± 1.55	4.66 ± 1.21	4.40 ± 1.35	4.19 ± 1.11	.037
T2DM, n (%)	58 (15.6)	37 (21.4)	23 (27.1)	32 (41.6)	<.001
Fasting plasma glucose (mmol/L)	5.70 ± 1.62	6.02 ± 1.87	6.06 ± 1.65	6.16 ± 1.61	.039
Rs7396366					
GG	206 (55.4)	89 (51.4)	30 (35.3)	43 (55.8)	.009
GT	137 (36.8)	63 (36.4)	45 (52.9)	19 (24.7)	.003
TT	29 (7.8)	21 (12.1)	10 (11.8)	15 (19.5)	.018

Abbreviations: *OR* odds ratio, *CI* confidence interval

Age, BMI, Total cholesterol and fasting plasma glucose are expressed as the mean ± SD and were compared using the one-way ANOVA. Other data are expressed as frequencies and percentages and were compared using the χ2-test

To further explore whether rs7396366 TT genotype can distinguish acute coronary syndrome (ACS) from SCAD, we performed the analysis and the results were shown in Table 6. However, no significant rs7396366 genotype difference is found between ACS and SCAD subgroups (Table 6).

**Table 6:** Association analysis of rs7396366 genotypes between ACS and SCAD cases

Genotypes	SCAD cases (*n* = 282)	ACS cases (*n* = 53)	OR (95% CI)	*P*	Multiple adjusted OR ^a^ (95% CI)	*P*
	*n* (%)	*n* (%)				
Rs7396366						
GG	136 (48.2)	26 (49.1)	1.03 (0.58–1.86)	.912	1.10 (0.60–2.04)	.755
GT	105 (37.2)	22 (41.5)	1.20 (0.66–2.17)	.556	1.25 (0.67–2.33)	.484
TT	241 (14.5)	5 (9.4)	0.61 (0.23–1.63)	.322	0.49 (0.18–1.36)	.172
Additive model			0.64 (0.23–1.76)	.525	0.47 (0.18–1.38)	.170
Dominant model			0.97 (0.54–1.74)	.912	0.91 (0.49–1.68)	.755
Additive model: TT vs. GG. Dominant model: (TT + GT) vs. GG						

^a^Logistic regression model, adjusted by age, sex, hypertension, T2DM, smoking, BMI, and fasting plasma glucose

The relationship of rs7396366 polymorphism and other risk factors between the three subgroups and controls is demonstrated in Table 7. Association is estimated in terms of odds ratio, 95% CI, and *P* values. The analysis indicated that age, sex, T2DM, fasting plasma glucose level, and especially rs7396366TT genotype were significant risk factors for three-vessel CAD (*P* < 0.001; *P* < 0.001; OR 3.85, 95% CI 2.26–6.56, *P* < 0.001; *P* = 0.023; OR 2.86, 95% CI 1.45–5.65, *P* = 0.005, respectively).

**Table 7:** The rs7396366 polymorphism and other risk factors in 1-, 2-, and 3-vessel lesion patients compared with controls

Variables	One-vessel disease		Two-vessel disease		Three-vessel disease	
	OR (95% CI)	*P*	OR (95% CI)	*P*	OR (95% CI)	*P*
Age	–	<.001	–	<.001	–	<.001
Sex	1.17 (0.81–1.67)	.406	2.42 (1.47–3.99)	<.001	2.49 (1.47–4.20)	<.001
BMI	–	.703	–	.343	–	.727
Smoking	1.00 (0.67–1.50)	.997	1.39 (0.84–2.30)	.230	1.62 (0.97–2.71)	.073
Hypertension	1.86 (1.26–2.75)	.002	2.48 (1.43–4.30)	.001	1.67 (0.99–2.85)	.054
Total cholesterol (mmol/L)	–	.848	–	.215	–	.018
T2DM	1.47 (0.93–2.33)	.115	2.01 (1.15–3.50)	.018	3.85 (2.26–6.56)	<.001
Fasting plasma glucose (mmol/L)	–	.040	–	.066	–	.023
Rs7396366(TT)	1.63 (0.90–2.96)	.112	1.58 (0.74–3.38)	.280	2.86 (1.45–5.65)	.005
Rs7396366 (TT vs. GG)	1.68(0.91–3.10)	.103	2.37 (1.05–5.35)	.048	2.48 (1.23–5.01)	.014

Abbreviations: *OR* odds ratio, *CI* confidence interval

Age, BMI, total cholesterol and fasting plasma glucose were compared using the Student *t* test. Other data were compared using the χ2-test

## Discussion

In this hospital-based case-control study, we confirmed an association between rs7396366 polymorphism, closest to the AP2A2 gene, and a higher risk of CAD and its severity based on a Chinese population. These findings are useful for early risk assessment and severity prediction of CAD.

Recently, a role for AP2A2 in hematopoietic stem cell activity was identified in adult mouse and embryonic 14.5-day old fetal liver cells [23]. More importantly, Buroker et al. found that compared with the DMSO-treated cells, an increase in AP2A2 activity is apparent in cardiomyocytes treated for 24 h with Wy14643 (a PPAR activator) [24]. All these data point to the possibility that AP2A2 plays a remarkable role in energy metabolism and lipid transport and is potentially associated with nerve and cardiac disease.

Based on the rapid progress of GWAS, several genetic variants of SNPs are considered to be associated with human diseases [25–27]. The AP2A2 gene has also been seen as an underlying risk gene for the pathogenesis of CAD [24]. A recent study [19] has demonstrated genetic overlap between CAD, C-reactive protein, and plasma lipids and have reported that the rs7396366 variant closest to the AP2A2 gene and the rs2526378 variant closest to the BZRAP1 gene were two suggested CAD susceptibility loci. Moreover, several studies reported the potential association between AP2A2 gene variants and asthma, wheeze in childhood and adolescence [28], and chronic bronchitis [29], and BZRAP1 variants and Alzheimer’s disease [30]. However, further studies concerning the association between the two SNPs and CAD have not been conducted. According to our results, rs7396366 TT genotype in CAD patients was significantly higher than in the controls. Subjects with rs7396366 TT genotype or T allele were more vulnerable to CAD than those with the GG genotype or G allele. Unfortunately, rs2526378 showed no such results, which may partly owe to the circumscription of sample size and thus needs further study.

In our study, age, sex, hypertension, and T2DM, which are all well-known risk factors for CAD, were also significantly different between CAD patients and non-CAD controls. A large prospective cohort study revealed that the incidence of new cardiovascular disease continued to increase after age 80 [31]. Similarly, a recent study showed that individuals > 65 years of age experience higher risk-adjusted hazards for major adverse cardiac events for non-obstructive, one-, and two-vessel disease, with similar event rates for three-vessel or left main artery disease compared with those < 65 years of age [32]. Interestingly, a study of Danish nonagenarians and centenarians demonstrated that although the individual risk for disability rose with age, disability in the population was not increased during the ninth decade of life [33]. One explanation for this is that everybody dies eventually; various causes of death competing in old people lead to further paradoxes [34]. Sex differences in the incidence, morbidity, and mortality from CAD are well documented [35, 36]. Substantial evidence shows that men, compared with women, experience a higher incidence and prevalence of CAD. The reason may partially be explained by their sex-based propensity to engage in higher-risk behaviors like smoking or excessive alcohol consumption [37]. According to an interaction model theory conducted in the present study, a male carrier of rs7396366 GT/TT genotypes has a higher risk for CAD.

Hypertension plays an important role in the development of CAD. When blood pressure increases, mechanical pressure, impaired endothelial function, and vascular active substances interact with each other to prompt coronary artery intimal injury, vascular wall hypertrophy, lipid deposition, atherosclerotic plaque formation, and the final CAD stage. T2DM is a major risk factor for CAD [38], and the cardiovascular system is particularly susceptible to the biological perturbations caused by this disease. Many patients die from T2DM-related cardiovascular complications [39]. In the current study, both T2DM and fasting plasma glucose were significantly associated with the severity of CAD.

### Limitations

First, the sample size of our hospital-based retrospective study is relatively small, so the persuasiveness of the results might be influenced. Second, selection bias is an inevitable factor in the study. Third, the constitution of the control group is noteworthy, because these non-CAD controls have more risk factors for CAD than the general population has. Fourth, the severity of CAD is not only related to the number of lesions, but also to other factors, such as stenosis degree and total scores, myocardial fractional flow reserve and so on. Therefore, “severity” is a general concept. In this study, to simplify the concept, the number of lesions is used to reflect the severity of CAD. Fifth, left main coronary artery lesions are not involved in the study due to its tiny proportion. Sixth, the precise pathogenic mechanism underlying the variants of rs7396366 contributing to CAD has not been determined and needs further study. Last but not least, because all the participants are Chinese, extending the results to a wider population is debatable. However, our study does provide a valuable perspective for further studies focusing on clarifying the association between AP2A2 gene variants and increased risk of CAD.

## Conclusions

In this hospital-based case-control study, we found that TT genotype of rs7396366 polymorphism of the AP2A2 gene may enhance the risk of CAD and reflect the severity of CAD. The rs7396366 TT genotype might be considered a potential risk marker to predict CAD and its severity. Nevertheless, due to the complexity of CAD, the role of AP2A2 gene in the formation of CAD remains to be further investigated.

## Additional file

Additional file 1: Flow chart. (DOCX 74 kb)

## Abbreviations

ACS: Acute coronary syndrome
AP2: Adaptor related protein complex 2
AP2A2: Adaptor related protein complex 2 alpha 2 subunit
BMI: Body mass index
BZRAP1: Benzodiazepine receptor-associated protein 1
CAD: Coronary artery disease
CI: Confidence interval
CVDs: Cardiovascular diseases
FPG: Fasting plasma glucose
GPCRs: G-protein coupled receptors
GWAS: Genome-wide association studies
NSTEMI: Non-ST-segment elevation myocardial infarction
OR: Odds ratio
SCAD: Stable coronary artery disease
SNPs: Single nucleotide polymorphisms
STEMI: ST-segment elevation myocardial infarction
T2DM: Type 2 diabetes mellitus

## References

[1] Clark H (2013). NCDs: a challenge to sustainable human development. Lancet.

[2] Roth GA, Johnson C, Abajobir A, Abd-Allah F, Abera SF, Abyu G (2017). Global, regional, and National Burden of cardiovascular diseases for 10 causes, 1990 to 2015. J Am Coll Cardiol.

[3] He J, Gu D, Wu X, Reynolds K, Duan X, Yao C (2005). Major causes of death among men and women in China. N Engl J Med.

[4] Chen WW, Gao RL, Liu LS, Zhu ML, Wang W, Wang YJ (2016). Outline of the report on cardiovascular diseases in China, 2014. Eur Heart J Suppl.

[5] Mangino M, Spector T (2013). Understanding coronary artery disease using twin studies. Heart.

[6] Smith JG, Newton-Cheh C (2015). Genome-wide association studies of late-onset cardiovascular disease. J Mol Cell Cardiol.

[7] Kathiresan S, Srivastava D (2012). Genetics of human cardiovascular disease. Cell.

[8] Chen X, Li S, Yang Y, Yang X, Liu Y, Liu Y (2012). Genome-wide association study validation identifies novel loci for atherosclerotic cardiovascular disease. J Thromb Haemost.

[9] Zeller T, Blankenberg S, Diemert P (2012). Genomewide association studies in cardiovascular disease--an update 2011. Clin Chem.

[10] Nelson CP, Goel A, Butterworth AS, Kanoni S, Webb TR, Marouli E (2017). Association analyses based on false discovery rate implicate new loci for coronary artery disease. Nat Genet.

[11] Schmid SL (1997). Clathrin-coated vesicle formation and protein sorting: an integrated process. Annu Rev Biochem.

[12] Collins BM, McCoy AJ, Kent HM, Evans PR, Owen DJ (2002). Molecular architecture and functional model of the endocytic AP2 complex. Cell.

[13] McMahon HT, Boucrot E (2011). Molecular mechanism and physiological functions of clathrin-mediated endocytosis. Nat Rev Mol Cell Biol.

[14] Kadlecova Z, Spielman SJ, Loerke D, Mohanakrishnan A, Reed DK, Schmid SL (2017). Regulation of clathrin-mediated endocytosis by hierarchical allosteric activation of AP2. J Cell Biol.

[15] Galiegue S, Jbilo O, Combes T, Bribes E, Carayon P, Le Fur G (1999). Cloning and characterization of PRAX-1. A new protein that specifically interacts with the peripheral benzodiazepine receptor. J Biol Chem.

[16] Chardenot P, Roubert C, Galiegue S, Casellas P, Le Fur G, Soubrie P (2002). Expression profile and up-regulation of PRAX-1 mRNA by antidepressant treatment in the rat brain. Mol Pharmacol.

[17] Bucan M, Abrahams BS, Wang K, Glessner JT, Herman EI, Sonnenblick LI (2009). Genome-wide analyses of exonic copy number variants in a family-based study point to novel autism susceptibility genes. PLoS Genet.

[18] Pinto D, Pagnamenta AT, Klei L, Anney R, Merico D, Regan R (2010). Functional impact of global rare copy number variation in autism spectrum disorders. Nature.

[19] Desikan RS, Schork AJ, Wang Y, Thompson WK, Dehghan A, Ridker PM (2015). Polygenic overlap between C-reactive protein, plasma lipids, and Alzheimer disease. Circulation.

[20] Khanna M, Cao W, Zirvi M, Paty P, Barany F (1999). Ligase detection reaction for identification of low abundance mutations. Clin Biochem.

[21] Xie F, Chen Z, Ding Z, Ma G (2013). A novel major histocompatibility complex locus confers the risk of premature coronary artery disease in a Chinese Han population. Mol Biol Rep.

[22] Xiao Z, Xiao J, Jiang Y, Zhang S, Yu M, Zhao J (2006). A novel method based on ligase detection reaction for low abundant YIDD mutants detection in hepatitis B virus. Hepatol Res.

[23] Ting SB, Deneault E, Hope K, Cellot S, Chagraoui J, Mayotte N (2012). Asymmetric segregation and self-renewal of hematopoietic stem and progenitor cells with endocytic Ap2a2. Blood.

[24] Buroker NE, Huang JY, Barboza J, Ledee DR, Eastman RJ, Reinecke H (2012). The adaptor-related protein complex 2, alpha 2 subunit (AP2alpha2) gene is a peroxisome proliferator-activated receptor cardiac target gene. Protein J.

[25] Hindorff LA, Sethupathy P, Junkins HA, Ramos EM, Mehta JP, Collins FS (2009). Potential etiologic and functional implications of genome-wide association loci for human diseases and traits. Proc Natl Acad Sci U S A.

[26] Dupuis J, Langenberg C, Prokopenko I, Saxena R, Soranzo N, Jackson AU (2010). New genetic loci implicated in fasting glucose homeostasis and their impact on type 2 diabetes risk. Nat Genet.

[27] Matarin M, Brown WM, Scholz S, Simon-Sanchez J, Fung HC, Hernandez D (2007). A genome-wide genotyping study in patients with ischaemic stroke: initial analysis and data release. Lancet Neurol.

[28] Arathimos R, Suderman M, Sharp GC, Burrows K, Granell R, Tilling K (2017). Epigenome-wide association study of asthma and wheeze in childhood and adolescence. Clin Epigenetics.

[29] Lee JH, Cho MH, Hersh CP, McDonald MLN, Crapo JD, Bakke PS, and on behalf of the COPDGene and ECLIPSE Investigators (2014). Genetic susceptibility for chronic bronchitis in chronic obstructive pulmonary disease. Respir Res.

[30] Jun GR, Chung J, Mez J, Barber R, Beecham GW, Bennett DA (2017). Transethnic genome-wide scan identifies novel Alzheimer's disease loci. Alzheimers Dement.

[31] Driver JA, Djousse L, Logroscino G, Gaziano JM, Kurth T (2008). Incidence of cardiovascular disease and cancer in advanced age: prospective cohort study. BMJ.

[32] Nakazato R, Arsanjani R, Achenbach S, Gransar H, Cheng VY, Dunning A (2014). Age-related risk of major adverse cardiac event risk and coronary artery disease extent and severity by coronary CT angiography: results from 15 187 patients from the international multisite CONFIRM study. Eur Heart J Cardiovasc Imaging.

[33] Christensen K, McGue M, Petersen I, Jeune B, Vaupel JW (2008). Exceptional longevity does not result in excessive levels of disability. Proc Natl Acad Sci U S A.

[34] Strandberg TE (2008). Cardiovascular disease and cancer in very old age. BMJ.

[35] Go AS, Mozaffarian D, Roger VL, Benjamin EJ, Berry JD, Blaha MJ (2014). Heart disease and stroke statistics--2014 update: a report from the American Heart Association. Circulation.

[36] Deo R, Albert CM (2012). Epidemiology and genetics of sudden cardiac death. Circulation.

[37] Spence JD, Pilote L (2015). Importance of sex and gender in atherosclerosis and cardiovascular disease. Atherosclerosis.

[38] Fox CS, Golden SH, Anderson C, Bray GA, Burke LE, de Boer IH (2015). Update on prevention of cardiovascular disease in adults with type 2 diabetes mellitus in light of recent evidence: a scientific statement from the American Heart Association and the American Diabetes Association. Circulation.

[39] Kovacic JC, Castellano JM, Farkouh ME, Fuster V (2014). The relationships between cardiovascular disease and diabetes: focus on pathogenesis. Endocrinol Metab Clin N Am.

