# Supplementary material for: A genetic variant near adaptor-related protein complex 2 alpha 2 subunit gene is associated with coronary artery disease in a Chinese population
Source: BMC Cardiovasc Disord. 2018 Aug 7;18:161. doi: 10.1186/s12872-018-0905-2 (PMC6081916; doi:10.1186/s12872-018-0905-2)
Supplement: Supplementary file 1 — Flow chart. (DOCX 74 kb) [file 12872_2018_905_MOESM1_ESM.docx]

Flow chart:

Statistical analysis: SPSS software version 19.0

Continuous variables: *t* test

Categorical variables: chi-square test

Multiple regression analysis

STEMI

Stable CAD

NSTEMI

Controls (excluded using cardiac catheterization)

References

Conclusions

Discussion

Results

Clinical variables: age, sex, BMI, hypertension, diabetes, TChol, FPG…

Two SNPs genotype determination: PCR-LDR strategy

Cardiac catheterization results

Clinical classification

3-vessel lesion

2-vessel lesion

1-vessel lesion

CAD cases (diagnosed using cardiac catheterization)

Participants
